# Hip fracture types in men and women change differently with age

**DOI:** 10.1186/1471-2318-10-12

**Published:** 2010-03-09

**Authors:** David A Tanner, Marita Kloseck, Richard G Crilly, Bert Chesworth, Jason Gilliland

**Affiliations:** 1 Graduate Program in Health and Rehabilitation Sciences, Faculty of Health Sciences, University of Western Ontario, London, Ontario, Canada; 2 Division of Geriatric Medicine, Parkwood Hospital, 801 Commissioners Road East, London, Ontario, N6C 5J1, Canada; 3 Department of Geography, University of Western Ontario, London, Ontario, Canada

## Abstract

**Background:**

Hip fractures are expensive and a frequent cause of morbidity and mortality in the elderly. In most studies hip fractures have been viewed as a unitary fracture but recently the two main types of fracture (intertrochanteric and subcapital) have been viewed as two fractures with a different etiology and requiring a different approach to prevention. The relative proportion of intertrochanteric fractures increases with age in women. In previous studies no particular pattern in men has been noted. In this study, we explored changes in the relative proportion of the two fracture types with age in the two genders.

**Methods:**

Patients of 50 years and older, with a diagnosis of hip fracture, discharged from two local acute care hospitals over a 5 year period (n = 2150) were analyzed as a function of age and gender to explore the relative proportions of intertrochanteric and subcapital fractures, and the change in relative proportion in the two genders with age.

**Results:**

Overall, for the genders combined, the proportion of intertrochanteric fractures increases with age (p = .007). In women this increase is significant (p < .001), but in men the opposite pattern is observed, with the proportion of intertrochanteric fractures falling significantly with age (p = .025).

**Conclusions:**

The pattern of hip fractures is different in men and women with aging. It is likely that the pattern difference reflects differences in type and rate of bone loss in the genders, but it is conjectured that the changing rate and pattern of falling with increasing age may also be important. The two main hip fracture types should be considered distinct and different and be studied separately in studies of cause and prevention.

## Background

Hip fractures are regarded as the most common severe type of fall-related injury among older adults and the most serious of the osteoporotic fractures because of their high morbidity, mortality and impairment in quality of life [1,2]. As the risk of hip fracture increases dramatically with age, it is a widely held view that the number of hip fractures will rise substantially as the population of Canada and the United States continues to grow older. Many studies have grouped hip fractures as a homogeneous condition, though there are two major anatomic types: intracapsular fractures (cervical or subcapital hip fractures) of the femoral neck and extracapsular hip fractures of the intertrochanteric region (pertrochanteric fractures). As the two major sites have a dissimilar composition of bone, the trochanteric region having a greater proportion of trabecular bone [3], it has been suggested that the etiology of each fracture may in fact differ and investigating hip fractures as a single entity may obscure risk factors and occurrence patterns. Thus those with intertrochanteric fractures tend to have lower bone density and more vertebral fractures suggesting they are more osteoporotic, although this finding is not totally consistent [4-7]. Beyond research which has shown that advancing age is more strongly associated with risk of intertrochanteric fractures than subcapital fractures [8], evidence for other such differences between the fracture populations remains largely unexplored, especially in recent years [4,5,7,9]. Karagas et al. (1996) found a rising proportion of intertrochanteric fractures in white women but not in white men, or blacks of either gender, and Bjorgul et al. (2007), likewise, found a rise in women but not in men [10]. The current study's objectives were to further explore, among a cohort of men and women with hip fracture, the relative proportion of intertrochanteric and subcapital fractures and how the proportion changes with age in the two genders.

## Methods

Secondary database analyses of de-identified hospital discharge abstracts from the catchment area of the two acute care hospitals in the city of London, Ontario, Canada (approximately 500,000 inhabitants) was conducted to examine the distribution of anatomic hip fracture types as a function of age and gender. Older adults were categorized by three traditional age strata; 'young' old (65-74 years), 'middle' old (75-84 years) and 'old' old (85+ years) to identify individuals in different stages of frailty. Current population-based definitions define frailty, for individuals living in developed countries, as beginning around the age of 85 years [11]. As hip fracture rates are known to increase exponentially from age 50 years and the most recent national literature reports hip fracture rates for individuals 50 years and older [12], an additional age stratum (50-64 years) was included in the analysis. All hip fractures were captured regardless of the subsequent outcome, including death of the patient.

### Study population and data source

Hospital discharge data as reported to the Canadian Institute for Health Information (CIHI) Discharge Abstract Database (DAD) on hip fracture admissions for individuals 50 years of age and older were analyzed for the period 2002 to 2006 inclusive. The DAD is a national database (with the exception of Quebec) containing information on all acute care hospital admissions which has been validated and shown to be of high quality [13]. The CIHI database provided information on all hospitalizations for the region. Hip fractures were defined according to the International Statistical Classification of Diseases and Related Health Problems, Tenth Revision, Canada (ICD-10-CA) as either subcapital fractures (S72.0-S72.091) or intertrochanteric fractures (S72.1-S72.191). Only those records with hip fracture as the most responsible diagnosis for the length of hospital stay were included. Exclusions were used to identify a homogeneous cohort of older patients who sustained a hip fracture through non-malignant mechanisms in an attempt to restrict analyses to fractures associated primarily with osteoporosis. Excluded were records of hip fracture patients <50 years of age, those indicated to have either malignant neoplasm (C00-D09) and/or motor vehicle related external cause of injury (V01-V99) codes in any diagnosis field, and duplicated records indicating a transfer for the same episode of hip fracture. After all exclusions, 2150 (1595 women, 555 men) hip fractures were left for analysis.

### Statistical analysis

Associations between age, gender and fracture type were explored. Analysis of the proportion of hip fracture types across the age groups and across gender was conducted using the Chi-Square method in SAS. This was followed by logistic regression to test for a significant interaction between age and gender on the likelihood of sustaining one fracture type compared to the other.

## Results

**Figure 1:**
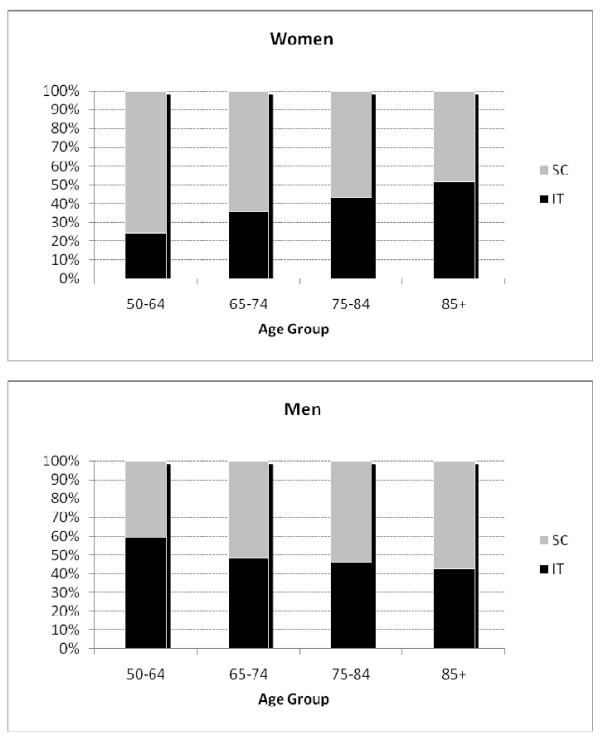
**The relative proportions of intertrochanteric (IT) and subcapital (SC) fractures by age in men and women**.

For all patients, age and fracture type were significantly related, with the proportion of intertrochanteric fractures increasing from 41.5 to 50 percent across age groups (p = 0.007). The proportion of the two types of hip fracture across the age categories for men and women is shown in Figure 1. A significant interaction was found by logistic regression analysis (p < 0.001), the relative proportion of the two types of hip fracture changing with age in a different manner in the two genders. In women the proportion of the hip fractures which occurred at the intertrochanteric site rises significantly with age across the four age groups (p < .001) whereas the proportion of intertrochanteric hip fractures among men decreases with age (p = .025). The absolute numbers are provided in Additional File 1. The mean age of women with intertrochanteric fractures is significantly older than those with subcapital fractures (83.9 ± 8.03 SD vs. 81.1 ± 9.23 SD; p < .001) whereas men with intertrochanteric fractures are younger than men with subcapital fractures (77.5 ± 11.02 SD vs. 79.3 ± 9.99 SD; p = .05).

## Discussion

Our data are consistent with those of Karagas et al. (1996) in showing a rise in the proportion of intertrochanteric fractures in women with increasing age, but not in men. Likewise, in a study of a Norwegian population, Bjorgul and Reikeras (2007) found an increasing tendency to intertrochanteric fractures relative to subcapital fractures in women with increasing age, but no pattern in men. We find, in men, a small but significant increase in subcapital fractures with aging. In women the proportion of intertrochanteric fractures rises from 24% in the youngest group to almost 52% in the oldest group, while in men it falls from 59% to 42% although most of the change occurs early, with little change after 65. Why the situation should be different for men and women is obscure and the change across the different ages is perplexing. Although there is evidence that the loss of trabecular and cortical bone with age may differ between men and women, the significance of this is unclear [14] but the rising proportion of intertrochanteric fractures in women may reflect greater trabecular bone loss with age in women. In the various studies where the prevention of hip fracture with treatment, usually with bisphosphonates, has been demonstrated, the study population has tended to be relatively young (generally around 70) and, by selection, usually with quite severe osteoporosis as shown by the presence of spinal fractures. This might leave one with the impression that hip fractures in the younger age groups are associated with osteoporosis, and hence more likely to be of intertrochanteric type, but the present data raises doubts about this, particularly in women, where the fracture less associated with osteoporosis, the subcapital, is dominant. Most people with fractures do not, in fact, have a bone mineral density consistent with osteoporosis [15,16]. This does raise the question of whether, even in younger women, falling, and not osteoporosis, is the major cause of hip fracture and identifying those with osteoporosis for preventive treatment may have only modest overall benefit.

Hip fractures are common and costly in terms of system expense and personal loss of independence. They represent a complex phenomenon, much more than the simple loss of bone mass with age. Equally important, and perhaps even more so with increasing age, is the tendency to fall and to fall in a different way [17]. The fall onto the greater trochanter is a phenomenon of old age. While it has been shown that the lateral fall onto the greater trochanter is particularly difficult to protect oneself during [18-20], the loss of multitasking ability and distraction by other activities that characterizes the oldest population members makes both preventing the fall and protecting oneself from injury even more difficult [21]. It appears that a fall onto the greater trochanter may generate enough force to break any hip [22]. It is also likely that the size of the target zone within which a strike will generate enough force to break the hip, increases as the fragility of the bone increases. It is known that the patients who suffer an intertrochanteric fracture have more osteoporosis, as shown by lower bone mineral density and more vertebral fractures [7,23]. It can be postulated that the fall onto the greater trochanter will produce a fracture of the intertrochanter site if the bones are fragile. The intertrochanteric region seems to absorb the force rather like the crumple zone of a car, and prevents the force being passed along to the neck of the femur. However, if the trabecular bone is strong, the force may be transferred to the neck of the femur which may then fracture. The greater loss of bone in women than in men may dispose the former to more intertrochanteric fractures with age. In addition, femoral neck length, greater in men, may be relevant as it tends to be associated with an increased risk of subcapital fracture, but this is not likely to change with age [24,25].

With increasing age, the fall becomes more common and fracturing may be more a reflection of the falling rather than thin bones, although the rising proportion of intertrochanteric fractures speaks to the rising prevalence of osteoporosis, at least in women. It is likely that falling dictates the prevalence of hip fracture, while the bone strength dictates who is more likely to fracture and the nature of the fracture that occurs.

It would have been of interest to present the actual rates of fracture for the two genders and all age groups, but we were unable to do so because of lack of precision regarding the catchment areas of the hospitals and hence uncertainty regarding the denominator. Our data, therefore, cannot address the absolute risk of hip fracture at different ages in the two genders but simply the relative risk of one type of hip fracture compared to the other.

## Conclusions

Our data show a change in the ratio of intertrochanteric versus subcapital fractures as a function of age with a different pattern in men and women. It is suggested that studies of hip fracture occurrence and prevention should treat hip fractures as two distinct fractures and report data for the two fracture types separately.

## Competing interests

The authors declare that they have no competing interests.

## Authors' contributions

DAT participated in conceptualization of the study, collected the data, and participated in data analyses. MK participated in conceptualization of the study, guided data collection, participated in data analyses and revised the manuscript. RGC participated in conceptualization of the study, guided data collection, participated in data analyses and drafted the manuscript. BC participated in design of the study, performed statistical analyses and participated in revising the manuscript. JG participated in conceptualization of the study. All authors have read and approved the final manuscript.

## Pre-publication history

The pre-publication history for this paper can be accessed here:

http://www.biomedcentral.com/1471-2318/10/12/prepub

## Supplementary Material

Additional file 1 **2009 Hip Fracture Types Table S1**. Number of hip fractures by type, sex and age strata in the city of London, ON, Canada 2002-2006. Click here for file
