# Supplementary material for: Hip fracture types in men and women change differently with age
Source: BMC Geriatr. 2010 Mar 9;10:12. doi: 10.1186/1471-2318-10-12 (PMC2850897; doi:10.1186/1471-2318-10-12)
Supplement: Additional file 1 — 2009 Hip Fracture Types Table S1. Number of hip fractures by type, sex and age strata in the city of London, ON, Canada 2002-2006. [file 1471-2318-10-12-S1.DOC]

# Additional File 1

Fracture Type

Sex Age Strata (years) Subcapital Intertrochanteric

50-64 56 (76%) 18 (24%)

65-74 116 (64%) 65 (36%)

Women 75-84 356 (57%) 271 (43%)

85+ 345 (48%) 368 (52%)

Mean age (SD) 81.1 (9.23) 83.9 (8.03)

50-64 30 (41%) 43 (59%)

65-74 42 (52%) 39 (48%)

Men 75-84 127 (54%) 109 (46%)

85+ 95 (58%) 70 (42%)

Mean age (SD) 79.3 (9.99) 77.5 (11.02)
